# Pharmaceutical Management of Secondary Hyperparathyroidism and the Role of Surgery: A 5-Year Retrospective Study

**DOI:** 10.3390/medicina60050812

**Published:** 2024-05-15

**Authors:** Christina Sevva, Dimitrios Divanis, Ariti Tsinari, Petros Grammenos, Styliani Laskou, Stylianos Mantalobas, Eleni Paschou, Vasiliki Magra, Periklis Kopsidas, Isaak Kesisoglou, Vassilios Liakopoulos, Konstantinos Sapalidis

**Affiliations:** 1 3rd Surgical Department, University General Hospital of Thessaloniki “AHEPA”, School of Medicine, Faculty of Health Sciences, Aristotle University of Thessaloniki, 1st St. Kiriakidi Street, 54621 Thessaloniki, Greece; stelaskou@gmail.com (S.L.); steliosmantalobas@yahoo.gr (S.M.); valiamagra@gmail.com (V.M.); ikesis@hotmail.com (I.K.); sapalidiskonstantinos@gmail.com (K.S.); 2 2nd Department of Nephrology, University General Hospital of Thessaloniki “AHEPA”, School of Medicine, Faculty of Health Sciences, Aristotle University of Thessaloniki, 1st St. Kiriakidi Street, 54621 Thessaloniki, Greece; ddivanis@gmail.com (D.D.); arititsi@gmail.com (A.T.); liakopul@otenet.gr (V.L.); 3 Department of Anesthesiology, University General Hospital of Thessaloniki “AHEPA”, School of Medicine, Faculty of Health Sciences, Aristotle University of Thessaloniki, 1st St. Kiriakidi Street, 54621 Thessaloniki, Greece; gr_petros@hotmail.com; 4 School of Medicine, Faculty of Health Sciences, Aristotle University of Thessaloniki, 1st St. Kiriakidi Street, 54621 Thessaloniki, Greece

**Keywords:** secondary hyperparathyroidism, calcimimetics, cinacalcet, etelcalcetide, parathyroidectomy, CKD

## Abstract

*Background and Objectives:* Secondary hyperparathyroidism (SHPT) poses a common condition among patients with chronic kidney disease (CKD) due to the chronic stimulation of the parathyroid glands as a result of persistently low calcium levels. As a first option for medical treatment, vitamin D receptor analogs (VDRAs) and calcimimetic agents are generally used. Apart from cinacalcet, which is orally taken, in recent years, another calcimimetic agent, etelcalcetide, is being administered intravenously during dialysis. *Materials and Methods:* In a 5-year retrospective study between 2018 and 2023, 52 patients undergoing dialysis were studied. The aim of this study is to highlight the possible effects and/or benefits that intravenously administered calcimimetic agents have on CKD patients. A total of 34 patients (65.4%) received cinacalcet and etelcalcetide while parathormone (PTH) and calcium serum levels were monitored on a monthly basis. *Results:* A total of 29 out of 33 patients (87.9%) that received treatment with etelcalcetide showed a significant decrease in PTH levels, which rose up to 57% compared to the initial values. None of the included patients needed to undergo parathyroidectomy (PTx) due to either extremely high and persistent PTH levels or severe side effects of the medications. It is generally strongly advised that parathyroidectomies should be performed by an expert surgical team. In recent years, a significant decrease in parathyroidectomies has been recorded globally, a fact that is mainly linked to the constantly wider use of new calcimimetic agents. This decrease in parathyroidectomies has resulted in an important decrease in complications occurring in cervical surgeries (e.g., perioperative hemorrhage and nerve damage). *Conslusions:* Despite the fact that these surgical complications cannot be easily compared to the pharmaceutical side effects, the recorded decrease in parathyroidectomies is considered to be notable, especially in cases of relapse where a difficult reoperation would be considered based on previously published guidelines.

## 1. Introduction

Parathyroid glands are small endocrine glands located in the posterior lateral surface of the thyroid gland. Their color is characterized as yellow–brown and their size varies, around approximately 5 mm × 4 mm × 2 mm with a weight of 20–40 mg. Many anatomical varieties have been described, mainly about the size and location of the glands. These differences arise from the embryological origin of the parathyroid glands. According to the global literature, the vast majority of the population, accounting for around 80–85% of people, appear to have four parathyroid glands. These are located on the posterior surface of the upper and lower lobes of the thyroid gland [1,2,3].

Parathyroid glands can be located inside the thyroid capsule. The two inferior parathyroid glands show the most anatomic variety regarding their location, as they can be either inside the thymus gland, the mediastinum, the paraesophageal area or the carotid sheath [1,2,4]. All normal parathyroid glands are supplied by the inferior thyroid arteries. In around 10–15% of the population, superior parathyroid glands may also be supplied by the superior thyroid arteries. Typically, most parathyroid glands are located within 1 cm from the point where the inferior thyroid artery meets the recurrent laryngeal nerve [1,4].

The main role of these glands is to regulate calcium metabolism, which takes place at the bones, the kidneys and the intestine. Serum calcium levels normally range between 8.2 and 10.2 mg/dL. Homeostasis and maintenance of these levels within normal range is achieved via a complicated regression adjustment mechanism including serum calcium, vitamin D and PTH [3].

PTH is a peptide consisting of 84 amino acids and is produced by the parathyroid glands. Normal PTH values are 10–65 pg/mL. It has a joint action both in the kidneys and in the bones, enhancing the reabsorption of calcium from the kidneys, while blocking the reabsorption of phosphorus. Moreover, it can cause an increase in the production and secretion of active vitamin D, thus causing an increase in the absorption of calcium from the bowel [5,6]. Calcitonin, on the other hand, is another hormone that is produced by the parafollicular cells of the thyroid gland. It has opposite effects to PTH, blocking calcium reabsorption by the kidneys [5].

While calcium homeostasis is regulated by the coordinated actions of PTH and calcitonin, sometimes, in cases where serum ionized calcium levels drop, an elevation in PTH secretion is observed together with low calcitonin secretion. High PTH levels promote both bone absorption and kidney calcium absorption, leading to a final increase in free calcium levels in the serum and normal ionized calcium levels [3,6].

Sometimes, this balance between serum calcium and PTH levels is disorganized, mostly due to the hypersecretion of PTH. As a result, a condition named hyperparathyroidism can occur, which can be further categorized to primary (PHPT), secondary (SHPT) and tertiary (THPT) hyperparathyroidism. In PHPT, uncontrolled secretion of PTH is observed, usually due to the presence of a single or multiple parathyroid adenomas, or hyperplasia of all glands, and rarely due to parathyroid malignancy. SHPT is characterized by a rise in PTH secretion due to the constantly low serum ionized calcium levels that occur in patients with CKD. Finally, in some cases where patients experience chronic SHPT, parathyroid glands tend to become hyperplastic due to the constant stimulation from low calcium levels, thus becoming autonomous. This situation is THPT, and it mainly affects people undergoing extrarenal dialysis, mainly hemodialysis [3,7,8].

This manuscript focuses on SHPT and its possible therapeutic approaches. In this condition, there is an increase in PTH secretion due to the low ionized calcium levels on the serum. Low ionized levels occur as a result of either CKD complications or vitamin D deficiency of calcium malabsorption. Phosphorus levels tend to be high in patients with CKD. However, in cases where low calcium and high PTH levels occur due to either malabsorption, rickets or osteomalacia, phosphorus levels appear to be either within normal range or even reduced [3,8].

SHPT usually appears in dialysis patients or patients undergoing peritoneal dialysis due to the overall decrease in renal function. Low ionized calcium levels occur due to decreased phosphorus levels due to nephron loss, or inadequate hydroxylation of the 25-hydroxy-cholecalciferol on the 1-a position, in order to produce the active metabolite of vitamin D (active D3 vitamin). Therefore, a decrease in intestinal absorption together with bone resistance to the effects of PTH and increase in calcitonin levels take place. In general, the glomerular filtration rate (GFR) decreases as CKD progresses, and thus, regulating mechanisms are more and more incapable of maintaining calcium and phosphorus levels. An increase in PTH levels occurs early in the progression of CKD, specifically from stage 3B, and this increase becomes even more noticeable as CKD progresses, leading to established SHPT [9].

Continuous simulation of the parathyroid tissue due to low calcium levels in patients with CKD can lead to the appearance of four specific patterns affecting the parathyroid glands: diffuse hyperplasia, primary nodular diffuse hyperplasia, nodular hyperplasia and finally, simple nodular gland [10].

Management of CKD can prove to be very challenging due to all involved parameters. First of all, high phosphorus levels must be lowered by using phosphorus binders, such as calcium carbonate and calcium citrate, or non-calcium-based binders, such as hydrochloric sevelamer, which has now been replaced by carbonate sevelamer, sucroferric oxyhydroxide and lanthanum carbonate [11]. Aluminum-based phosphorus binders were also widely used in the past; however, their use has been restricted due to high aluminum toxicity in the CNS, liver and other organs [12,13]. Vitamin D deficiency should also be addressed by administering cholecalciferol (D3) or ergocalciferol (D2). Vitamin D receptor agonists (VDRAs) are also used. Calcitriol, alphacacidol and doxercalciferol are non-selective agonists, while selective agonists include paricalcitol, paricalcitol, maxacalcitol and phalecalcitriol [11,14,15,16]. However, the most currently effective option in controlling PTH levels seem to be calcimimetic agents. Through allosteric activation of the calcium-sensing receptor (CaSR), calcimimetic agents lower PTH and phosphorus levels [10,11,17]. Cinacalcet is the most effective and commonly used oral first generation calcimimetic, while etelcalcetide is an intravenously administered second generation agent, first approved in 2016 in Europe, that seems to be 30% more effective than cinacalcet in reducing PTH levels [10,17,18].

Sometimes, however, even though all medical resorts have been tried, PTH levels remain uncontrollable. Surgical treatment then remains the only possible and reasonable option. All global guidelines concur that total parathyroidectomy with the excision of 3.5 parathyroid glands and autotransplantation of the remaining healthy gland in the sternocleidomastoid or the brachioradialis muscle is the gold standard procedure [11,19,20,21,22].

## 2. Materials and Methods

The aim of this retrospective study is to present the study and analysis of the effect of an intravenously administered calcimimetic drug, etelcalcetide, on patients with SHPT. The main objective of the study is to underline the possible contribution of etelcalcetide to the considerable reduction in parathyroidectomies, as well as the reoperation rate in this category of patients. Patients with CKD are prime candidates for presenting with SHPT due to the general decrease in renal function. Calcimimetics pose a new category of drugs that have been widely administered for the past 20 years to patients undergoing dialysis in order to handle hyperparathyroidism. However, due to poor compliance on orally administered calcimimetic agents, new intravenously administered agents have been introduced. Etelcalcetide is a new second generation calcimimetic agent, available only for inpatient use in our country. Through this study, the authors aim to underline the effects of this substance in controlling high PTH levels. By showing this, it is evident that if SHPT can be managed via pharmaceutical means, patients with CKD will be less likely to undergo a highly specialized surgical operation, that of total parathyroidectomy.

This five-year retrospective study was conducted in patients from the 2nd Department of Nephrology of the University General Hospital of Thessaloniki AHEPA, who underwent hemodialysis during the time period between 1 June 2018 and 1 June 2023, Sample characteristics included the following: that patients were over 18 years old; they were systematically receiving a specific VDRA–paricalcitol and phosphorus binders (either ferric oxyhydroxide or sevelamer carbonate). More specifically, inclusion criteria included adult patients over 18 years old, who were further divided into five subgroups (18–30, 31–45, 46–60, 61–75, and 76–90), in order to facilitate statistical analysis. A minimum of a 6-month time period undergoing dialysis was decided on in order for enough data to be acquired. Other inclusion criteria included a diagnosis of SHPT without a previous parathyroidectomy and patients undergoing weekly hemodialysis in an artificial kidney machine. Exclusion criteria included patients who underwent parathyroidectomy and/or kidney transplant, while patients who underwent peritoneal dialysis were excluded. Also excluded from the study were patients appearing with Long QT Syndrome (LQTS) together with prelabour and postpartum patients. Finally, hospitalized patients were not included, so that no patient would present hypoalbuminemia and would not require to calculate the corrected calcium. The sample size arose after taking into consideration all patients who underwent dialysis in the Nephrology Department who met the inclusion criteria.

Patients underwent 3 sessions of hemodialysis per week (12 per month). Etelcalcetide was administered either to patients that had never received a first generation calcimimetic or to patients that were initially treated with first generation calcimimetics and then switched to second generation calcimimetics under clinicians’ judgment. A weekly follow-up with monitoring of parathormone and calcium serum levels was set; however, for this particular statistical analysis, only data from a monthly base were taken into consideration. Biochemical blood samples were always obtained prior to dialysis session; therefore, before the patients received etelcalcetide, all samples were handled and analyzed by the same laboratory. Finally, 52 of this department’s patients met all set criteria and were included in the sample of this study (Table 1). The study design and access to data were approved and granted by the hospital’s Institutional Review Board.

## 3. Statistical Methods–Data Analysis

For the statistical analysis, the Analysis ToolPak of Excel by Microsoft Office Professional Plus 2019^®^ was used. The general characteristics of the studied population were as follows: from the sum of 52 patients, 35 (67.3%) were men and 17 (32.7%) were women. The age of the patients ranged between 33 and 89, with a mean age of 67 years and an SD (Standard Deviation) of 11.8 years. As seen clearly in Table 1, the vast majority of patients (29) accounting for 55.8% of the sample group belong to the age group ranging between 61 and 75 years old. During these five years, 16 patients (30.7%) passed away due to complications connected with hemodialysis. At the beginning of the inclusion on the first hemodialysis session, the majority of the patients showed a significantly affected value of parathormone. More specifically, 48 out of the 52 patients (92.3%) showed a significant increase in parathormone, averaged at 221 pg/mL (SD = 136.4). At the same time, 13 patients (25%) showed significant hypocalcemia, with serum calcium < 8.2 mg/dL, while the average value of blood calcium for the total of the patients at the beginning of their inclusion was 8.6 mg/dL (SD = 0.62) (Table 2). Given the fact that our sample size is 52, and considering a 95% confidence level, this study’s results come with a margin of error of 8.15%, calculated with the CI (confidence interval) Formula.

## 4. Results

**Figure 1:**
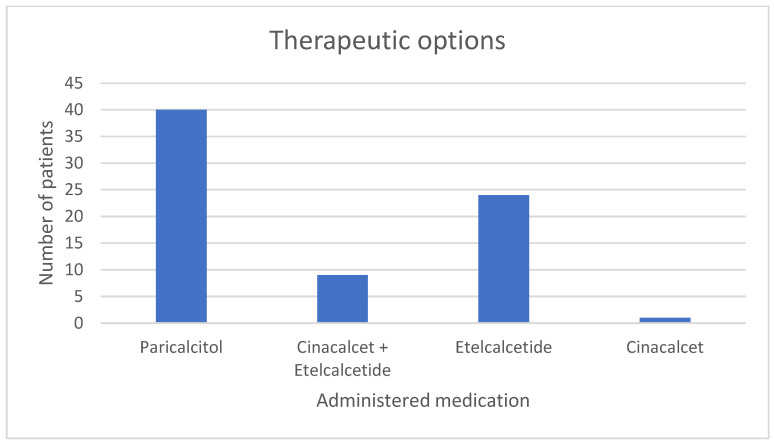
Therapeutic options that were followed in this specific protocol: 40 patients received paricalcitol (VDRA), 9 patients received cinacalcet and then they switched to etelcalcetide, 24 patients received solely etelcalcetide and 1 patient received only cinacalcet.

During dialysis, 40 patients (76.9%) received paricalcitol, a selective VDRA, in order to handle the tendency to chronic hypocalcemia, via correcting vitamin D deficiency. In patients with persistent SHPT and non-manageable PTH levels, administration of calcimimetics–either cinacalcet (oral) or etelcalcetide (intravenous)–was considered necessary. Specifically, 9 patients (17.3%) received both substances, initially cinacalcet and later switched to etelcalcetide, 24 patients (46.1%) received exclusively etelcalcetide and 1 patient (1.9%) received exclusively cinacalcet (Figure 1).

Up until 2016, the only available approved calcimimetic was cinacalcet, which originally received approval in Europe and the United States of America in 2004 [18]. Cinacalcet is a first generation calcimimetic agent, which reacts with the membrane receptor of CaSR, increasing its sensitivity to extracellular calcium and thus reinforcing the signaling pathway and finally reducing secretion of PTH. The substance has 80% renal excretion and a half-life time of 30–40 h. For that reason, it is administered daily per os, and levels and dosage should be adjusted every 2–4 weeks. Available dosage formulations include tablets of 30 mg, 60 mg or 90 mg and the ideal dosing range is estimated between 30 and 180 mg daily [23,24] In several metanalyses that have been published, it seems that cinacalcet has a >30% reduction in initial PTH values [18,25]. Patient compliance to this therapy, though, is considered an important factor that cannot be guaranteed, as it tends to be lower and lower as time progresses, due to the common side effects on the gastrointestinal system (GIS), including severe nausea, vomiting and diarrhea. This is also the reason why etelcalcetide is mostly preferred among physicians in dialysis patients. Patient compliance does not pose an issue in this case, as the substance is being administered during dialysis session. Other side effects of cinacalcet include headache, very low or very high values of blood pressure, muscle spasms, symptomatic or asymptomatic hypocalcemia and, rarely, QT prolongation [18,26].

Surprisingly enough, none of the patients of the group reported any side effects and clinicians did not observe any unexpected reactions after administrating etelcalcetide to the group of patients. Moreover, all nine patients who had been initially treated with cinacalcet and then switched to etelcalcetide reported a better tolerance in the second generation calcimimetic agent and no disorders from the gastrointestinal system.

Since 2016 though, management of SHPT received completely new dimensions as etelcalcetide received approval in Europe for inpatient use solely. The same approval by the FDA came one year later, in 2017 [18,24,27]. This synthetic second generation peptide calcimimetic agent is a CaSR agonist with exclusively renal excretion and a half-time life of >7 days. It is intravenously administered via a bolus infusion three times a week, during or at the end of each dialysis session. Available dosage formulations include single–dose vials of 2.5 mg, 5 mg or 10 mg, while the ideal dosing range is considered to be between 2.5 and 15 mg per dialysis session [24]. Patient compliance is now guaranteed as the substance and dosage is controlled exclusively by physicians. Side effects of etelcalcetide also include minor symptoms from the GIS (nausea, vomiting, diarrhea); however, they appear less frequently and in a smaller magnitude than in cinacalcet [25]. Specifically, patients included in this study did not report any side effects during the administration of etelcalcetide. Due to better patient compliance and a general better biological action mechanism of the substance, patients under etelcalcetide show a more important decrease in PTH levels compared to patients under cinacalcet [17].

**Figure 2:**
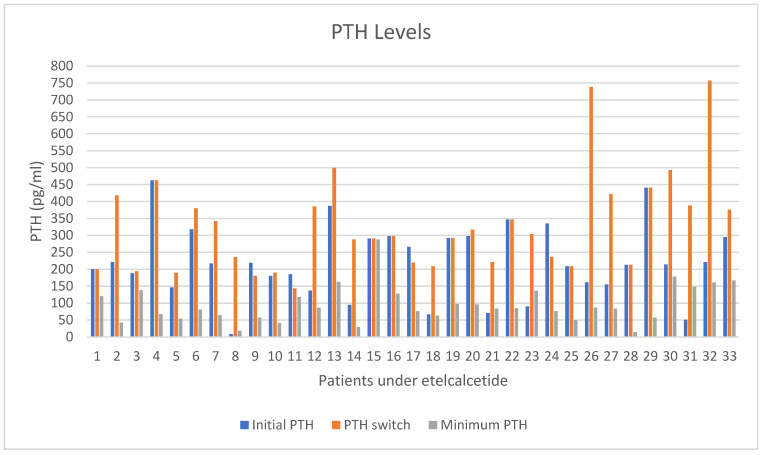
Record of the transition of PTH levels in 33 patients who received etelcalcetide intravenously. Initial PTH: PTH levels upon beginning of dialysis. PTH switch: PTH levels when administration of etelcalcetide begun. Minimum PTH: the lowest levels of PTH accomplished after treatment with etelcalcetide.

In this protocol, a total of 33 patients received systematically intravenous etelcalcetide. In these patients, PTH and serum calcium were being measured monthly and minimum PTH levels together with maximum reduction rate in every patient were calculated. A total of 29 out of 33 patients (87.9%) showed a reduction in PTH levels. The mean value of minimum recorded PTH levels was 91 pg/mL in a range of 18–178 pg/mL (SD = 53.9). After running a one-tailed distribution, two-sample equal variance *t*-test, the mean total PTH reduction rate in patients of this study under etelcalcetide was 57% with a *p* value of 0.0000008783, which is statistically significant. In Figure 2, the range of PTH value for every single patient receiving etelcalcetide can be observed, thus showing that in every patient, a lower value of PTH than the value measured at the time that etelcalcetide was firstly administered was achieved (Figure 2).

From this record, an important decrease in PTH levels is observed. This reduction, shown after treatment with an intravenously administered calcimimetic in patients with CKD and SHPT undergoing dialysis, also has an eminent effect on the number of parathyroidectomies performed on the group of patients with those specific characteristics.

## 5. Discussion

For the past 20 years, parathyroidectomy, as a therapeutic solution for SHPT, has remained a controversial topic. The original guidelines of KDOQI 2003 (Kidney Disease Outcomes Quality Initiative) of the National Kidney Foundation (NKF) had set a strong recommendation for performing parathyroidectomy in patients with a PTH value of >800 pg/mL and even with a PTH value of >500 pg/mL in some cases where severe calciphylaxis was also present [28]. The recommended surgical operation is total parathyroidectomy, which consists of bilateral exploration of the neck and removal of 3.5 parathyroid glands and autotransplantation of the macroscopically-appearing healthier gland, usually inside the sternocleidomastoid muscle [11,19,20,21,22]. After undergoing parathyroidectomy, patients have shown a dramatic decrease in PTH levels, a 95% decrease in pathological fractures and a reduction in soft tissue calcifications within 6–8 weeks, together with an improvement in clinical symptoms, mainly in pain and pruritus. Surprisingly enough, there is a decrease in the overall mortality danger from all causes ranging from 15% to 57% [29,30]. 

Six years later, the KDIGO 2009 (Kidney Disease: Improving Global Outcomes) guidelines made a recommendation for parathyroidectomies in patients under dialysis with stages 3-5D CKD who did not respond to pharmaceutical treatment (type 2B recommendation). Parathyroidectomy is recommended to be performed by an expert surgeon. A specific cut-off value for PTH, however, is not specified [31]. In 2010, the NFK, evaluating the above-mentioned KDIGO guidelines, released the KDOQI 2010 guidelines where parathyroidectomy is also recommended in patients who resist in treatment with medication and who moreover present with a lot of side effects from those treatments. They underline, however, that not all patients are eligible for surgery and that total performance status of the patient and perioperative hazard should be taken into serious consideration [32].

The most recent guidelines regarding parathyroidectomy in SHPT are included in the KDIGO 2017 guidelines. Overall, the general recommendation for parathyroidectomy has not been altered compared to the 2009 guidelines; however, the use of pharmaceutical treatment using VDRAs and calcimimetic agent, either as a monotherapy or using a combination of medication, has been reinforced [11]. Towards the same direction, the KDOQI 2017 guidelines also support the use of calcimimetic agents, while questioning the current place of parathyroidectomies [33].

Data from a retrospective study with a total of 606 patients with CKD and SHPT that were handled with parathyroidectomy between 1976 and 2009 showed that 15% of patients undergoing dialysis are going to need parathyroidectomy after 5–10 years of continuous dialysis sessions, while this percentage rises to 38% after 20 years of continuous dialysis [10,29,34]. Comparing data from this retrospective study with our study, several conclusions are derived. Although the authors are aware of the significantly smaller sample of the present retrospective study, we should note that there are 23 patients (44.2% of the sample) that have been under dialysis for at least 5 years and none of them have undergone parathyroidectomy. Moreover, there are another 10 patients (19.2%) undergoing dialysis for more than 10 years, none of whom have needed to undergo parathyroidectomy based on PTH levels (Table 1).

In this observed difference, perhaps the time periods in which data were collected played a role. More specifically, the retrospective study of the 606 patients refer to a time period prior to 2010, where international guidelines from kidney organizations were either non-existent or lacked enough evidence on surgical management of SHPT. On the contrary, this present study was conducted after 2017, where all of the most recent guidelines are applied, and all newer medication are administered. A very important role in this differentiation is also played by the approval and wide use of cinacalcet in 2004, which brought a decrease in performed parathyroidectomies firstly in Japan, and then in a smaller range in the USA, Canada, Europe, Australia and New Zealand [35].

The previously presented comparison regarding the need for performing parathyroidectomies in addition to the evolution and update of the guidelines regarding management of SHPT leads to the conclusion that pharmaceutical treatment using VDRAs and calcimimetic agents–especially intravenous ones–have managed a better and more efficient control of PTH levels. This also has an effect on the number of performed parathyroidectomies, which are being significantly restricted. These operations are, nevertheless, considered to be challenging operations due to complications that can derive from the bilateral neck exploration and cases of reoperation (such as post-operative hemorrhage, temporary or permanent damage of the recurrent laryngeal nerve and the external branch of superior laryngeal nerve, surgical site infections and hungry bone syndrome) and that should be exclusively performed in any case by an expert surgical team [36].

These complications can be further categorized into two subgroups: the ones relating to the wound and nearby structures and the ones relating to intraoperative conditions and findings. Therefore, complications of the first group include post-operative hemorrhage leading to the formation of a hematoma that will need a surgical incision and drainage in order to find the source of the bleeding before the hematoma expands and airway patency is compromised. Another complication is seroma and infections of the wound, with the latter occurring usually after the presence of a hematoma. Finally, skin tethering and keloid formation are two more complications affecting the external wound. The second group includes nerve damage, mostly on the recurrent laryngeal nerve and the external branch of superior laryngeal nerve, leading to dysphonia and even airway obstructions if nerve injury is bilateral. These damages can be temporary or permanent depending on the type of injury (temporary after thermal or other injury, permanent after dissection of the nerves). Systematic complications are also a postoperative issue, with the most common being temporary or permanent hypoparathyroidism, persistent hypocalcemia, hungry bone syndrome and Horner syndrome [36,37].

By conducting this study, the authors have attempted to show that SHPT can be managed satisfyingly enough, achieving acceptable PTH levels in patients with end-stage CKD by using new second generation calcimimetic agents, while tampering with patient compliance and minimizing side effects deriving from first generation calcimimetic agents. Parathyroidectomy is not by any means discouraged or contraindicated but should be left as a resource in very young patients with good performance status, high estimated life expectancy and pharmaceutically uncontrolled PTH levels.

## 6. Conclusions

SHPT consists of a common condition among patients with end-stage CKD, thus making the management of PTH levels a challenge. The past year’s therapeutic options have been enriched, making calcimimetic agents an important addition to the already widely used VDRAs. Although cinacalcet showed very satisfying results in the immediate reduction in PTH levels, it seems to lack in patient compliance due to severe side effects from the GIS. This patient compliance seemed highly likely to be solved in 2019 by the constantly increasing use of etelcalcetide, which ensured full patient compliance, fewer side effects, and higher reduction in PTH levels compared to cinacalcet. This present study is aiming to present the results from an admittedly small group, thus involving limitations regarding generalization of the results. Nevertheless, it indicates the global tendency toward fewer parathyroidectomies, underlining the possible superiority of newer intravenous calcimimetic agents versus the technically challenging parathyroidectomies and neck reoperations.

## Figures and Tables

**Table 1:** Overall characteristics of the patients consisting of the sample included in the study.

Patient Characteristics	Number (*n*)	Percentage (%)
Number of patients	52	100%
Sex:		
• Male	35	67.3%
• Female	17	32.7%
Medical conditions		
• Hospitalized	0	0%
• Non hospitalized	52	100%
Age groups:		
• 18–30	0	0%
• 31–45	3	5.8%
• 46–60	11	21.1%
• 61–75	29	55.8%
• 76–90	9	17.3%
Years on dialysis:		
• 0–5	29	55.8%
• 6–10	13	25%
• 11–15	7	13.4%
• 16–20	1	2%
• >20	2	3.8%

**Table 2:** Distribution of patients based on PTH levels and calcium levels at the time of inclusion to the study.

		Number of Patients	Percentage of Patients
PTH Value	Calcium Levels		
High PTH		48	92.3%
	High Calcium	0	0%
	Normal Calcium	39	75%
	Low Calcium	13	25%
Normal PTH		2	3.8%
	High Calcium	0	0%
	Normal Calcium	2	100%
	Low Calcium	0	0%
Low PTH		2	3.8%
	High Calcium	0	0%
	Normal Calcium	2	100%
	Low Calcium	0	0%

Normal value range for serum PTH: 10–65 pg/mL; normal value range for Calcium: 8.2–10.2 mg/dL.

## Data Availability

The raw data supporting the conclusions of this article will be made available by the authors on request.
